# α‐CsPbI_3_ Quantum Dots ReRAM with High Air Stability Working by Valance Change Filamentary Mechanism

**DOI:** 10.1002/smtd.202400514

**Published:** 2024-08-06

**Authors:** Da Eun Lee, In Hyuk Im, Ji Hyun Baek, Kyung Ju Kwak, Seung Ju Kim, Tae Hyung Lee, Jae Young Kim, Ho Won Jang

**Affiliations:** ^1^ Department of Materials Science and Engineering Research Institute of Advanced Materials Seoul National University Seoul 08826 Republic of Korea; ^2^ Department of Electrical and Computer Engineering University of Southern California Los Angeles CA 90089 USA; ^3^ Advanced Institute of Convergence Technology Seoul National University Suwon 16229 Republic of Korea

**Keywords:** all‐inorganic halide perovskite, CsPbI_3_, quantum dots, resistive switching, valence change mechanism

## Abstract

The current memory system is facing obstacles to improvement, and ReRAM is considered a powerful alternative. All‐inorganic α‐CsPbI_3_ perovskite‐based ReRAM working by electrochemical mechanism is reported, but the electrochemically active electrode raised difficulty in long‐term stable operation, and bulk α‐CsPbI_3_ device can not show resistive switching behavior with an inert metal top electrode. Herein, by making the α‐CsPbI_3_ into QDs and applying it to the device with inert Au as the top electrode, the devices working by valence change mechanism are successfully fabricated. The large surface‐to‐volume ratio made an abundant amount of iodine vacancies and facile migration of vacancies allowed the device to work by valence change mechanism. The devices show reliable electrical characteristics, 800 cycles endurance and retention for over 4 × 10^4^ s, and air stability for 1 month. This work demonstrates that applying the QDs can improve the stability and enable a new type of working mechanism in ReRAM.

## Graphical abstract

Making the α‐CsPbI3 into QDs and applying it to the device with inert Au as the top electrode result in the valence change mechanism‐based devices. The large surface‐to‐volume ratio makes an abundant amount of iodine vacancies and facile migration of vacancies allows the device to work by valence change mechanism.

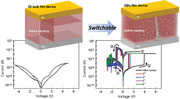

## 1 Introduction

ReRAM is one of the most promising nonvolatile memory devices to replace current memory systems, which are mainly dynamic random‐access memory (DRAM) and NAND flash memory. There is a large gap in memory hierarchy between these two types of memories.^[^ ^1^, ^2^ ^]^ Therefore, many researchers aspire for memristors that can conduct both functions, the fast computing realized by DRAM and long‐term storage realized by flash memories, enabling neuromorphic computing, and logic‐in‐memory applications.^[^ ^3^, ^4^, ^5^ ^]^ The answer to this aspiration can be emerging non‐volatile memory, typically magnetic RAM (MRAM), phase‐change RAM (PRAM), and resistive‐switching RAM (ReRAM). Among these new types of memories, ReRAM got attention because of its low power consumption, fast switching speed, and high integration density.^[^ ^1^, ^3^ ^]^

ReRAM has a simple structure of metal/semiconducting layer/metal. The semiconducting layer acts as the active layer, connecting and disconnecting the two metal electrodes by electrochemical metallization mechanism (ECM) or valence‐change mechanism (VCM). By applying a voltage with appropriate amplitude and polarity, the device stores data by reversibly changing between a low‐resistance state (LRS) and a high‐resistance state (HRS).^[^ ^4^, ^6^ ^]^ ReRAM based on various metal oxides and perovskite oxides were usually studied at the early stage of ReRAM. However, oxide‐based devices confronted many limitations, such as complicated components, and rigid films leading to poor flexibility and high‐temperature processing requirements.^[^ ^7^ ^]^ These limitations restricted ReRAM from commercialization and large‐scale production. To meet memory performance requirements for next‐generation computing system applications, other advanced materials to replace conventional oxide‐based materials should be studied.^[^ ^6^, ^8^ ^]^

Organometallic and all‐inorganic halide perovskites (HPs) with the formula ABX_3_, where A is an organic (ex. CH_3_NH_3_) or inorganic (ex. Cs, Rb) cation, B is a metal cation (ex. Pb, Sn), and X is a halide anion (ex. I, Br, or Cl), are considered as promising alternate materials for ReRAM.^[^ ^4^, ^9^ ^]^ They show many advantages over oxide‐based materials, such as simple fabrication, exotic properties such as facile majority carrier control, superior flexibility, tunable bandgaps, fast ion migration, and excellent optoelectronic characteristics.^[^ ^10^ ^]^ Recently, progress in HP‐based RS memory devices has been rapid because of their unique property of current–voltage hysteresis arising from fast ion migration.^[^ ^6^, ^10^, ^11^ ^]^ Resistive switching (RS) behaviors have been observed in various HPs such as organometallic (CH_3_NH_3_PbI_3_ and CH_3_NH_3_PbI_3‐_ *_x_* Cl*_x_*),^[^ ^12^, ^13^, ^14^ ^]^ all‐inorganic (CsPbI_3_^[^ ^15^, ^16^ ^]^ and Cs_3_Bi_2_I_9_^[^ ^17^, ^18^ ^]^) and 2D‐layered (BA_2_MA*_n_*_−1_Pb*_n_* I_3_ *_n_*_+1_ (BA = butylammonium, MA = methylammonium)) HPs.^[^ ^19^ ^]^ However, the organic part of the HP acts as a stumbling block to temperature endurance. Therefore, all‐inorganic HPs are considered more appropriate for stable devices. The resulting materials are lead‐based HPs, with A site possessed by Cs. However, only the CsPbBr_3_ are usually studied because of structural stability.^[^ ^20^ ^]^ However, CsPbBr_3_ raises toxic issues. Therefore, many researchers are searching for materials with no bromine element. CsPbI_3_ is one of them and is less studied because α‐CsPbI_3_ immediately turns into γ‐CsPbI_3_ in air. However, by making the material in nanometer‐sized quantum dots (QDs), the α‐CsPbI_3_ showed improved stability toward air.^[^ ^21^, ^22^, ^23^ ^]^ Based on this stability arising from the size of the material, we successfully fabricated Au(or Ag)/α‐CsPbI_3_ QDs/PEDOT:PSS/ITO devices. The device showed filamentary‐type RS behavior with bipolar switching characteristics. The RS behavior could be repeated even after 1 month of exposure to air, indicating superior stability to moisture and oxygen. To properly understand the mechanism of the device, the temperature‐dependent measurement was done and analyzed. These measurements confirmed the all‐inorganic halide perovskite ReRAM with high stability to air and temperature. Also, by changing the top electrode, the electrochemically active Ag, and inert Au, different RS behaviors were possible. These findings might be useful to further design the HPs‐based ReRAM for various purposes. This work will pave the way for QDs to be actively applied in the ReRAM field for improving endurance and stability.

## 2 Result and Discussion

### 2.1 Synthesis of α‐CsPbI3 Quantum Dots

**Figure 1:**
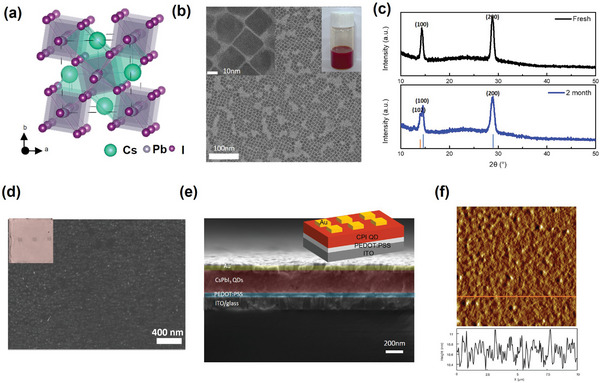
Memory device structure and characterization of α‐CsPbI_3_ quantum dots. a) Schematic illustration of α‐CsPbI_3_ perovskite phase with cubic structure. b) Transmission electron microscopy (TEM) image of synthesized QDs, inserted image on the left is TEM image with higher resolution and inserted on the right is the picture of actual solution showing the red color. c) XRD pattern of the perovskite QDs deposited on a glass substrate. The XRD on the top is the pattern measured immediately after synthesis and the bottom XRD pattern is measured after 2 months of exposure to air. d) Plane‐view SEM image of the QDs layer. The inserted image is the picture of the actual device. e) Cross‐sectional SEM image of the device in color and the inserted image is the schematic drawing of the device. f) Atomic force microscopy (AFM) image of the QDs layer.

The α‐CsPbI_3_ quantum dots (QDs) of the cubic structure were successfully synthesized by the hot‐injection method (**Figure** **1**a) according to a reported procedure.^[^ ^23^, ^24^ ^]^ The detailed synthesis steps can be found in the Experimental Section. The transmission electron microscopy (TEM) images of the α‐CsPbI_3_ QDs (Figure 1b) verify the uniform cubic morphology and the size of 5–16 nm (Figure S3, Supporting Information). The α‐CsPbI_3_ in bulk form were unstable in air, transforming into γ‐CsPbI_3_ with orthorhombic structure.^[^ ^15^ ^]^ However, α‐CsPbI_3_ could endure for 2 months in QDs.^[^ ^22^ ^]^ The obvious peaks in the X‐ray diffraction (XRD) measurement, indexed as (100) and (200) at 14° and 28° indicate the crystallinity and stability under moisture and air (Figure 1c). Using these α‐CsPbI_3_ QDs as a semiconducting layer, a ReRAM based on all‐inorganic halide perovskite was successfully fabricated, and switching characteristics were studied. The structure of the fabricated memory device is shown in Figure 1d,e. The thin PEDOT: PSS of 50 nm formed over ITO/glass helps QDs to be more adhesive to the underlying layer.^[^ ^25^ ^]^ The previously made α‐CsPbI_3_ QDs in hexane solution were spread over the PEDOT: PSS layer and formed a 250 nm thickness layer by annealing process at 150 °C. Au with 40 nm thickness acts as the top electrode. The thickness of each layer can be identified by a cross‐sectional scanning electron microscopy (SEM) image (Figure 1e) and a uniform QD layer is shown in a plane‐view SEM image (Figure 1d). The smooth film could also be verified by the atomic force microscope (AFM) image in Figure 1f. The measured RMS rougness was 1.217 nm which confirms the smoothness of the QDs film.

### 2.2 Electrical Measurement of HP QDs‐Based Device

#### 2.2.1 Electrical Characteristics of Au Top Electrode Device

**Figure 2:**
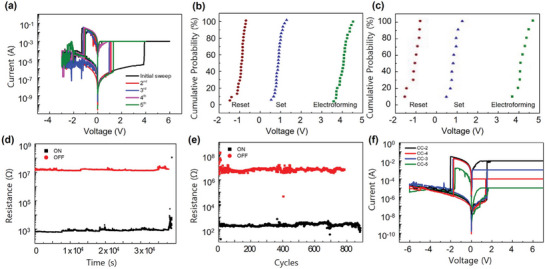
RS characteristics of Au/α‐CsPbI_3_ QDs/PEDOT: PSS/ITO devices. a) Series of *I–V* behaviors for the device. b) Cumulative probability of forming, setting, and reset voltage 25 different cells. The mean value (µ) for V_forming_ is 4.04 and the standard deviation (σ) is 0.25. For V_set_, µ = 0.86 and σ = 0.17. For V_reset_, µ = −0.96 and σ = 0.19. c) Cumulative probability of forming, setting, and reset voltage for 10 different devices. For V_forming_, µ = 3.97 and σ = 0.2. For V_set_, µ = 0.86 and σ = 0.2. For V_reset_, µ = −1.04 and σ = 0.25. d) Reversible RS endurance under continuous write/erase voltage pulses of +1.5 V, 10 ms pulse duration, and −2.5 V, 20 ms pulse duration at the read voltage of +0.05 V. e) Retention characteristics of LRS and HRS. f) Multilevel switching in *I–V* curves under the four different current compliance (CC = 10^−2^, 10^−3^, 10^−4^, and 10^−5^ A).

The RS characteristics of Au/α‐CsPbI_3_ QDs/PEDOT: PSS/ITO devices are shown in **Figure** **2**. The device exhibited filamentary and bipolar RS behavior. Figure 2a shows the series of current–voltage (*I–V*) hysteresis loop measured under direct current (DC) voltage sweep of 0 V → +4 V (+6 V) → 0 V → ‐3 V → 0 V, using a scan rate of 1 V·s^−1^. During the process, the PEDOT: PSS/ITO bottom electrode was grounded, and the voltage was applied to the Au top electrode.

The compliance current (CC) of 1 mA was set to prevent the degradation of the device during the positive voltage sweep where the SET process occurs. The cell required an electroforming process, indicating the filamentary switching behavior. The electroforming process occurred at +4.12 V, and the RESET process occurred at −1.28 V. During the subsequent DC sweep, the SET process occurred at +1.02 V and the RESET process at −1.14 V. The electroforming process is a typical RS behavior in filamentary type, which is not shown in an interface‐type RS.^[^ ^6^, ^26^ ^]^ The abrupt change in current indicates that the filament finally conducts the top and bottom electrodes. Once the filament is formed, the filament can be partially degraded and reformed by applying the appropriate voltage. Since forming the entire filament requires more power than rebuilding the partially degraded filament, the electroforming voltage is larger than the SET voltage.^[^ ^27^ ^]^ The abrupt change in resistance between the high resistance state (HRS) with ≈7 × 10^6^ Ω to the low resistance state (LRS) with ≈5 × 10^2^ Ω could be found throughout the repeated DC voltage sweep, meaning the reversible RS behavior. To confirm the operational reliability, electroforming, SET, and RESET voltage were measured over 25 different cells and 10 different devices and they are shown in Figure 2b,c. The results clearly show that the Au/α‐CsPbI_3_ QDs/PEDOT: PSS/ITO devices have excellent reliability; the variations between cells or devices were acceptable and the average values for cells and devices were almost the same. Under a reading voltage of +0.05 V, the HRS and LRS could be maintained for 4 × 10^4^ s, keeping the on/off ratio of ≈8 × 10^4^ (Figure 2d). The RS characteristics by alternating current (AC) voltage pulses were also measured. By this measurement, the endurance of the device could be verified. The device turned from HRS to LRS by applying the +1.5 V for 10 ms, and it turned back to the HRS state by applying −2.5 V for 20 ms with a reading voltage of +0.05 V. The reversible SET and RESET process could be conducted for 800 cycles (Figure 2e). Since the on/off ratio was over 10^4^, multilevel switching was expected for the device. Four different resistance states were possible (10^−5^, 10^−4^, 10^−3^, and 10^−2^ A) by modulating the compliance current in the SET process during the DC sweep (Figure 2f). The multiple resistance level has a great advantage for storage density because one cell can show various states without shrinking the physical size of the cell.^[^ ^28^ ^]^ The high storage density arising from the small cell size of the resistance‐based non‐volatile memory is one of the biggest advantages compared with traditional memory devices, and multilevel states^[^ ^16^, ^19^ ^]^ enhance the density of the memory even more, enabling to overcome the physical limitation of the current memory devices.

**Figure 3:**
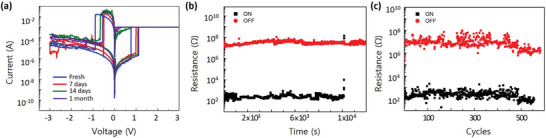
RS characteristics of Au/α‐CsPbI_3_ QDs/PEDOT: PSS/ITO devices after exposure in air a) Typical *I–V* curves for fresh, 7 days, 14 days, and 1‐month exposure to air. b) Retention characteristics of LRS and HRS after exposure to air for 2 weeks. c) Reversible RS endurance under continuous write/erase voltage pulses of +1.5 V, 10 ms pulse duration, and −2.5 V, 20 ms pulse duration at the read voltage of +0.05 V after exposure to air for 2 weeks.

By making the α‐CsPbI_3_ in QDs, the cubic phase showed fairly good stability over the air, confirmed by XRD after 2 months in the air. (Figure 1c) To confirm that the reliability of the material also applies to the α‐CsPbI_3_ in QDs‐based RS devices, the RS behavior was also measured over air‐exposed devices. The *I*–*V* curves were measured under a DC sweep of 0 V → +3 V → 0 V → −3 V→ 0 V, using a scan rate of 1 V·s^−1^ after 2 weeks and 1‐month exposure to air (**Figure** **3**a). Though some variations in the SET and RESET voltage are found, they are negligible and still showed hysteresis even after 1 month in air. To further find out the stability of the device, the retention and endurance were also measured for the device exposed to air for 2 weeks. The measurement condition was the same as the fresh sample. Compared to the fresh sample, the retention and endurance figure decreased. The HRS and LRS maintained for ≈10^4^ s (Figure 3b), which is one‐fourth of the fresh sample, and AC voltage cycles were possible for ≈550 times (Figure 3c), which is about two‐thirds of the fresh sample. However, this figure is still a noble figure for all‐inorganic HP‐based ReRAM.

#### 2.2.2 Working Mechanism of Au Top Electrode Device

**Scheme 1:**
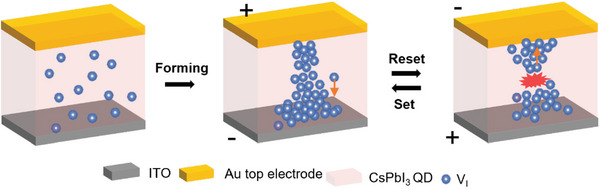
Schematic of mechanism in Au/α‐CsPbI_3_ QDs/PEDOT: PSS/ITO devices. By applying positive bias at the top electrode, iodine vacancies are repelled from the top electrode and pile up at the bottom electrode. When a number of vacancies are piled up, the top and bottom electrodes are connected by vacancies. When a negative bias is applied at the top electrode after the forming process, some vacancies forming the filament are attracted to the top electrode, resulting in the rupture of the conducting filament. Continuous set and reset processes occur by the formation and rupture of conducting filament under an electric field.

**Figure 4:**
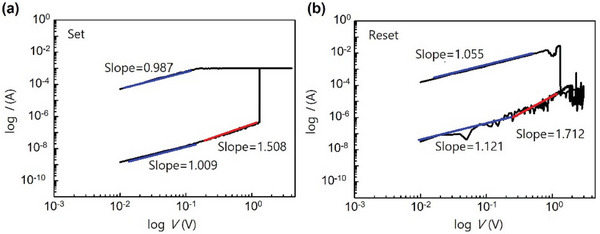
Conduction mechanisms of LRS and HRS. a,b) Double‐logarithm plot of the *I–V* characteristics of (a) SET process (b) RESET process.

Looking at the retention characteristic (Figure 2d), the LRS could not be maintained after ≈4 × 10^4^ s and the device returned to HRS after. It indicates that the filament is degraded and no longer connects the bottom and top electrodes. However, looking at the endurance characteristic, the failure occurs by complete degradation of the cell (Figure 2e). To understand this phenomenon, the conduction mechanism should be explained. Since the electroforming stage is shown in RS behavior, there is no doubt that the device works by filament.^[^ ^27^ ^]^ The filament is formed with iodine vacancies. By applying the positive voltage on the top electrode, the iodine vacancies are repelled to the bottom electrode and iodine anions drive toward the top electrode.^[^ ^29^, ^30^ ^]^ The vacancies piled up at the bottom electrode grow toward the top electrode, finally connecting the two electrodes through the active layer. When a negative voltage is applied on the top electrode, iodine vacancies, and anions move in opposite directions, partially degrading the filament, and this results in an HRS state (**Scheme** **1**). Retention failure can be explained by iodine vacancies going back to their original position by diffusion since there is no longer the coercive force to maintain the filament. The assumption that filament constituent is iodine vacancies can be explained from the thermodynamic point.^[^ ^50^ ^]^ From previous research, it is known that the formation of halide and A‐site vacancies (V_X_ and V_A_) is as easy as a pair of Schottky vacancies, maintaining charge neutrality of the lattice. That is because forming halide or A‐site vacancies requires less thermodynamic energy compared to point defects such as interstitials or antisites and B‐site vacancies. In the case of CsPbI_3_, the formation energy of iodine vacancies is smaller than Cs vacancies.^[^ ^15^ ^]^ Therefore, assuming that filaments are formed with iodine vacancies is reasonable. Since the filament is formed with iodine vacancies,^[^ ^31^ ^]^ the movement of vacancies and anions completely degrades the structure of halide perovskite as the stimuli become excessive. To further interpret the electrical transport and confirm the conduction mechanism, the *I–V* sweeps were replotted on a double‐logarithm scale (**Figure** **4**). The detailed equations about working mechanisms are described in Equation S1. During the SET process under 0 V → +3 V → 0 V, the initial device in HRS shows linear ohmic conduction (*I* ∝ *V*) with a slope of 1.009 (Figure 4a). However, as the applying voltage gets bigger, the space‐charge‐limited‐current (SCLC) conduction (*I* ∝ *V* ^2^) gets involved,^[^ ^32^ ^]^ which can be confirmed by a slope of 1.508. After the complete conversion into LRS, the slope of 0.987 indicates the ohmic conduction. During the RESET process under 0 V → ‐3 V → 0 V, the device in LRS shows ohmic conduction with a slope of 1.055 (Figure 4b). However, once the device is changed into HRS, there are no filaments so the conduction through the filament resulting in ohmic conduction is not possible. The SCLC is dominant at relatively high voltage because the external force is still large enough to influence light halide anion and vacancies. As the external force gets smaller, only the ohmic conduction occurs. It can be confirmed by the change in the slope in HRS, from 1.712 to 1.121. This indicates the VCM occurring in the Au/α‐CsPbI_3_ QDs/PEDOT: PSS/ITO device.

#### 2.2.3 Comparison of Electrical Properties between Ag and Au Top Electrode Device

**Scheme 2:**
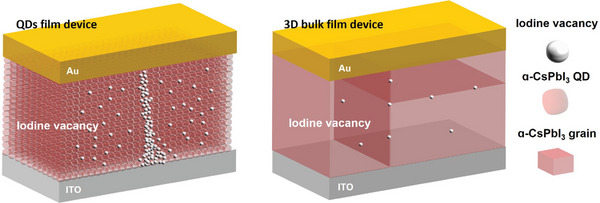
Schematic construction of the active layer, the one with QDs on the left and the bulk film on the right. Each component in the scheme is labeled on the right. Due to the large volume‐to‐surface ratio, an abundant amount of vacancies are generated in the QD film, while a small amount of vacancies in the 3D bulk film cannot generate the filament. It means that filament can be formed in QD film under abundant vacancies while the lack of vacancies blocks the formation of filament in bulk film.

We also fabricated the device with the structure of Ag/α‐CsPbI_3_ QDs/PEDOT: PSS/ITO. When active metal was used for the top electrode, the device showed different characteristics compared with an inert top electrode device. The SET voltage was +1.2 V, the RESET voltage was −1 V, and the electroforming voltage was +5.8 V, which is a bigger figure compared with the Au top electrode device (Figure S1a, Supporting Information). These figures were measured by applying the DC voltage sweep of 0 V → +2 V (+8 V) → 0 V → −2 V→ 0 V, using a scan rate of 1 V·s^−1^. The three multilevel resistance states were possible in this device with CC of 10^−2^ A, 10^−3^ A, and 10^−4^ A (Figure S1b, Supporting Information). However, the SET process was not possible with a compliance current of 10^−5^ A. Compared with a device with an inert metal top electrode, fewer multilevel states were possible. It was due to a smaller on/off ratio of 10^3^, resulting from more conducting HRS. By applying the AC voltage pulses, the endurance and retention of the device could be verified. The device turned from HRS to LRS by applying the +1.5 V for 10 ms, and it turned back to HRS by applying −2.0 V for 20 ms. The transition could be repeated for only ≈110 cycles, which is a smaller figure than the device with an inert metal top electrode exposed to air for 2 weeks (Figure S1c, Supporting Information). Also, the retention was only ≈2.5 × 10^3^ s (Figure S1d, Supporting Information). The electrical properties show that the device using an inert metal electrode shows much better RS behavior. The advantage of VCM is emphasized once again, which is the virtue of this research. This is due to the constituents of filaments. When the active metal, Ag in this case, is used as the top electrode, Ag easily dissolute to Ag cation. These cations move by external stimuli and metallic conducting filaments are formed and degraded by electrochemical reactions and thermal effects, respectively. Since the QD layer is an aggregation of QDs unlike the bulk film, the QD layer does not have big grains (**Scheme** **2**). When the relatively heavy and large Ag cations move through the small‐sized particles, the layer could be easily deformed, leading to degradation of the device which means short endurance cycles and retention time. By comparing the Ag and Au top electrode devices, the VCM is confirmed once more. Scheme 2 can also explain why the CsPbI_3_ QDs‐Au top electrode device showed novel electrical characteristics while the bulk CsPbI_3_‐Au device could not work as ReRAM.^[^ ^15^ ^]^ The innate abundant iodine vacancies exist in the QDs layer compared with bulk film,^[^ ^33^ ^]^ since the large surface‐to‐volume ratio contributes to the small activation energy for iodine turning into anion. The large surface‐to‐volume ratio can be clearly seen in the top‐view SEM image of the actual devices (Figure S2, Supporting Information). While there is a lack of halide vacancies in the bulk film, the abundant amount in the QDs layer makes it possible to form vacancy‐based filaments throughout the active layer. The merits of QDs‐based devices are emphasized once again. **Table** **1** shows that there is no CsPbI_3_ QDs‐based ReRAM working by VCM and supports the novelty of this device.^[^ ^36^, ^37^, ^38^, ^39^, ^40^, ^41^ ^]^

**Table 1:** Comparison of all‐inorganic halide perovskite‐based ReRAM based on CsPbBr_3_ and CsPbI_3_.

Structure	V_set_ [V]	V_reset_ [V]	ON/OFF ratio	Endurance [times]	Retention [s]	Ref.
ITO/PMMA/CsPbBr_3_ QDs/PMMA/Ag	+2.3 (w/o light) +1.1 (with light: 365 nm, 0.153 mW cm^−2^)	−2.7 (w/o light) −1.7 (with light: 365 nm, 0.153 mW cm^−2^)	≈10^5^	5000	4 × 10^5^	[20]
FTO/CsPbBr_3_ QDs/ZnO/Ni	−0.95	+0.71	≈10^5^	100	10^4^	[36]
ITO/CsPbBr_3_ QDs/Au	−2.4	+1.55	≈10^7^		10^3^	[37]
ITO/CsPbBr_3_ QDs/PMMA/Ag	+0.78	−1.12	≈10^2^	10^4^	10^5^	[38]
ITO/few layer black phosphorous nanosheets (FLBP)‐CsPbBr_3_ QDs heterostructure/Ag	+1.20	+1.16	10^7^	50	‐	[39]
ITO/PMMA:CsPbCl_3_ QDs/Al	−0.3	+2.6	2 × 10^4^	90	>10^4^	[40]
ITO/PEDOT:PSS/poly(N,N’‐bis‐4‐butylphenyl‐N,N’‐bisphenyl)benzidine (polyTPD)oleylguanidinium bromide(OGB)‐capped CsPbBr_3_ NCs/Ag	+1	−0.8	10^3^	5000	10^5^	[41]
Pt/CsPbI_3_/PMMA/Ag	+0.18	−0.1	>10^6^	300	‐	[15]
ITO/CsSnBr_3_/Au	+0.2	−0.15	≈10^5^	400	≈10^3^	[42]
Ag/CsSnCl_3_/ITO	+1.0	−1.1	≈10^2^	400	≈10^4^	[43]
Pt/CsSnBr_3_/Pt	+1.5	+0.9	10^2^	10^5^	>10^4^	[44]
Pt/Cs_2_AgBiBr_6_/ITO	+2	−2	500	–	1200	[45]
Al/CsBi_3_I_10_/ITO	+3.1	−2	10^3^	150	10^4^	[46]
Au/Rb_3_Bi_2_I_9_/Pt/Ti/SiO_2_/Si	+0.09	−0.3	10^6^	200	10^3^	[47]
Ag/PMMA/Cs_3_Cu_2_I_5_/ITO	+0.42	−0.53	10^2^	10^2^	>10^4^	[48]
Al/Cs_3_Cu_2_I_5_/ITO	+1.44	−0.55	≈65	200	10^4^	[49]
Au/α‐CsPbI_3_ QDs/PEDOT: PSS/ITO	+1.44	−0.55	8 × 10^4^	800	4 × 10^4^	This work

#### 2.2.4 Analysis of Working Mechanism by Temperature‐Dependent Tendency

**Figure 5:**
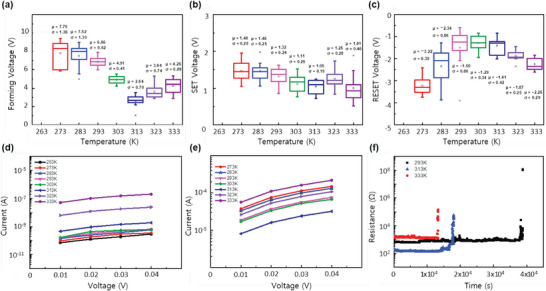
The RS behavior changes by altering the operation temperature from 273 to 333 K. a) Electroforming voltage change. b) SET voltage. c) RESET voltage. d) OFF current. e) ON current measured for low voltage pulse. f) the retention characteristic under 293, 313, and 333 K.

For a more detailed explanation, the electrical characteristics were conducted under various temperatures. The electroforming, SET, and RESET voltages were measured from 273 to 333 K. As the temperature increased up to 313 K, electroforming, SET, and RESET voltage all decreased. However, as the temperature increased more than 313 K, electroforming, and RESET voltage increased with temperature. The SET voltage also showed a slight increase (**Figure** **5**a,b,c). To understand this phenomenon, we further checked the ON, and OFF currents at different temperatures and retention characteristics of LRS at 293, 313, and 333 K (Figure 5d,e,f). ON current decreased as the temperature increased up to 313 K, and began to increase when the temperature became higher than 313 K (Figure 5e). Looking at Figure 5d, OFF currents showed a constant tendency to increase with temperature. The retention (Figure 5f) shows that the device could retain the LRS for up to 4 × 10^4^ s at a low temperature of 293 K, decreasing to 1.8 × 10^4^ s at 313 K, and finally to 1.2 × 10^4^ s at 333 K. These tendencies could be explained by the movement of the ions, causing the difference in the shape of the filaments.^[^ ^34^ ^]^ When the temperature is under 313 K, the mobility of ions mainly influences the conduction mechanism. Therefore, as the temperature increases, the ions move more easily and faster. The fast and facile movement of ions leads to a cluster of ions at the interface, not in the middle of the active layer. Thus, thin filaments are formed resulting in low current. However, as the temperature becomes >313 K, the collision of ions is not negligible. The collision between ions slows down the movement of ions and the number of ions reaching the interface decreases. In addition to the more active collision of ions with increasing temperature, the amount of Cs vacancies increases. As mentioned in Section 2.2.2, Cs vacancies and iodine vacancies are the most preferred defects formed in CsPbI_3_. Referring to the energy band of CsPbI_3_ confirms the assumption once again.^[^ ^51^ ^]^ As the temperature increases, that leads to slow movement of iodine vacancies because of electrical attraction. Therefore, ions meet in the middle of the active layer, forming thick filaments resulting in a higher current.^[^ ^33^, ^35^ ^]^ The off current, which the filament does not influence, shows a constant tendency with temperature, while the on current, which is influenced by filament, shows different tendencies at high and low temperatures. If the filament constituents were not iodine vacancies, the temperature tendency would have shown quite a different phenomenon. The retention characteristic further supported the movement of light iodine vacancies. The decreasing retention time with the temperature increase means the easier diffusion of vacancies under no external stimuli.

## 3 Conclusion

We successfully fabricated the α‐CsPbI_3_ QDs and devices with inert metal (Au) were fabricated for RS memory. While the bulk α‐CsPbI_3‐_based device did not show electrical hysteresis with inert metal as the top electrode, the α‐CsPbI_3_ QDs‐based device exhibits bipolar RS characteristics with high on/off ratio, multilevel data storage, long retention, and superior stability toward air. The small size of the QDs caused a large surface‐to‐volume ratio and made an abundant amount of iodine vacancies in the semiconducting layer. The device was able to work by VCM and showed superior stability to air, while the bulk film‐based device was not able to work by VCM. The performance is noticeable among all‐inorganic halide perovskite ReRAMs, especially compared with devices with VCM working mechanisms. While our current work has concentrated on the single‐device level of halide perovskite QDs‐based RS memory, there is a need to integrate them into arrays on a larger scale by incorporating selection devices or developing self‐rectifying devices. This research demonstrates the advantage of applying the QDs in the RS memory field, which can improve stability. It will raise aspiration to find new inorganic HP materials with high stability and help commercialization of ReRAM.

## 4 Experimental Section

### Materials

All chemicals were purchased from Sigma‐Aldrich and used as received unless otherwise noted. Oleic acid (OA, technical grade 90%), oleylamine (OAm, technical grade 70%), 1‐octadecene (ODE, technical grade 90%), cesium carbonate (Cs_2_CO_3_, 99.9%), methyl acetate (MeOAc, anhydrous 99.5%), lead iodide (PbI_2_, 99%, Alfa Aesar), n‐hexane (≥ 97.5%, Alfa Aesar), n‐octane (98+%, Alfa Aesar), and toluene (analytical reagent, 98%).

### Fabrication of RS Memory Device—*Synthesis of CsPbI_3_ QDs*

A total of 0.3 g of Cs_2_CO_3_ powder, 1.2 mL of OA, and 30 mL of ODE were loaded into a 100 mL three‐neck flask and degassed under vacuum at 120 °C for 60 min. The flask was filled with N_2_ and heated up to 150 °C until all Cs_2_CO_3_ reacted with OA to form Cs‐oleate. The Cs‐oleate solution in ODE was stored in N_2_ and kept at 100 °C for QD synthesis.

For the synthesis of CsPbI_3_ QDs, 0.5 g of PbI_2_ and 25 mL of ODE were loaded into a 100 mL three‐neck flask and degassed at 100 °C for 60 min under vigorous stirring. The flask was kept under constant N_2_ flow, followed by the injection with 2.5 mL of OA and 2.5 mL of OAm. The flask was degassed at 100 °C for 2 h followed by N_2_ flow, heating up to 165 °C.

Then, 4 mL of preheated Cs‐oleate in ODE (≈0.0625 m) was injected into the reaction mixture. The mixture turned dark red, and after 10 s, the flask was quenched by an ice water bath. The CsPbI_3_ QDs reaction liquor was loaded into three centrifuge tubes. Typically, 30 mL of MeOAc was added into 10 mL of QDs (QDs:MeOAc = 1:3 in v:v) and then centrifuged at 8000 RPM for 5 min. The precipitate was re‐dispersed with 3 mL of hexane, followed by adding 3 mL of MeOAc. The dispersion appeared cloudy and was immediately centrifuged at 8000 RPM for 3 min again. Finally, the QDs were re‐dispersed in 12 mL of hexane and centrifuged at 4000 RPM for 5 min to remove excess PbI_2_ and Cs‐oleate.

### Fabrication of RS Memory Device—*Fabrication of Memory Device*

The glass/ITO substrates were cleaned ultrasonically using acetone, isopropanol, and deionized water sequentially then treated with UV‐generated ozone for 15 min before making the thin PEDOT: PSS layer over it. The prepared precursor PEDOT: PSS solution was spin‐coated onto the substrates with a spin‐coating rate of 4000 rpm for 30 s and annealed at 150 °C for 15 min. CsPbI_3_ QDs (≈70 mg mL^−1^ in hexane) were spin‐coated on PEDOT: PSS layer at 3000 RPM for 40 s. 200 µL of MeOAc was loaded onto the QD film for 5 s to remove the surface ligands and spun at 2000 RPM for 20 s. This process was repeated three times to build up a thick QD film (≈250 nm). The QD film deposition processes were performed in an N_2_‐filled glovebox with a controlled temperature of 25 °C and relative humidity of <10%. Once the desired film thickness was achieved, the device was annealed for 15 min at 150 °C to aggregate quantum dot particles and make a dense film layer. Finally, the top electrode (Au or Ag) was deposited onto the HP QDs layer/PEDOT:PSS/ITO using e‐beam evaporation at a pressure of 1 × 10^−6^ Torr and a 50 µm × 50 µm patterned shadow mask at room temperature.

### Characterization

XRD measurements were recorded at room temperature in the 2θ range of 10–50° with a step size of 0.02° and a scan speed of 5° min^−1^, using an X‐ray diffractometer (BRUKER MILLER Co., D8‐Advance) with Cu Kα radiation (λ = 1.54056 Å). The HP QDs‐film surface images and cross‐sectional device images were obtained using a field‐emission scanning electron microscope (FE‐SEM, ZEISS, MERLIN Compact) with an in‐lens secondary electron detector operating at a 1 kV accelerating voltage. AFM (Park systems XE100) was used to determine the topography of the QD film formed by the spin coating method. The electrical properties were measured with an Agilent 4156C semiconductor analyzer and Tektronix AFG 3021C in the direct current voltage‐sweeping mode and alternating voltage pulse mode. The detailed size and morphology of quantum dots were taken by transmission electron microscope (JEOL Ltd., JEM‐2100F) operating at 200 kV.

### Statistical Analysis

All data were analyzed using Microsoft Excel (Microsoft), OriginPro 9.0 (OriginLab Co., Northampton, MA, USA), and MATLAB R2021b (MathWorks). The µ and σ values of V_forming_, V_set_, and V_reset_ are calculated by the normal distribution of electrical properties in different 10–25 cells.

## Conflict of Interest

The authors declare no conflict of interest.

## Supporting information

Supporting Information

## Data Availability

The data that support the findings of this study are available from the corresponding author upon reasonable request.
