# Supplementary material for: α‐CsPbI3 Quantum Dots ReRAM with High Air Stability Working by Valance Change Filamentary Mechanism
Source: Small Methods. 2024 Aug 6;9(1):2400514. doi: 10.1002/smtd.202400514 (PMC11740931; doi:10.1002/smtd.202400514)
Supplement: Supplementary file 1 — Supporting Information [file SMTD-9-2400514-s001.docx]

**Supporting information**

α-CsPbI_3_ Quantum Dots ReRAM with High Air Stability Working by Valance Change Filamentary Mechanism

*Da Eun Lee**^1^, In Hyuk Im^1^, Ji Hyun Baek^1^, Kyung Ju Kwak^1^, Seung Ju Kim^1, 2^, Tae Hyung Lee^1^, Jae Young Kim^1^, and Ho Won Jang*^, 1, 3^*

D. E. Lee, I. H. Im, J. H. Baek, K. J. Kwak, S. J. Kim, T. H. Lee, J. Y. Kim, Prof. H. W. Jang

^1^ Department of Materials Science and Engineering

Research Institute of Advanced Materials

Seoul National University

Seoul 08826, Republic of Korea

E-mail: hwjang@snu.ac.kr

S. J. Kim

^2^ Department of Electrical and Computer Engineering

University of Southern California

Los Angeles, CA 90089, USA

Prof. H. W. Jang

^3^ Advanced Institute of Convergence Technology

Seoul National University

Suwon 16229, Republic of Korea

Email: hwjang@snu.ac.kr

**
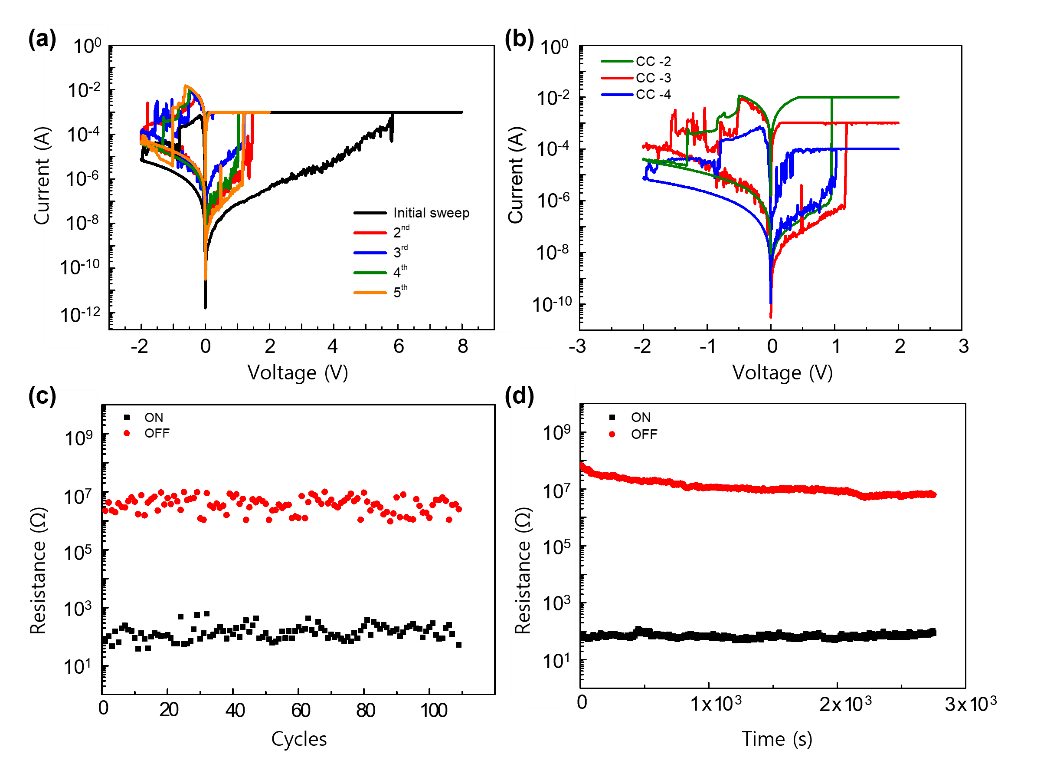
**

1. RS cahracteristics with active electrode(Ag)

Figure S1 RS characteristics of Ag/α-CsPbI_3_ QDs/PEDOT: PSS/ITO devices. (a) Series of *I–V* behaviors for the device. (b) Multilevel switching in *I-V* curves under the four different current compliance (CC = 10^−2^, 10^−3^, and 10^−4^). (c) Reversible RS endurance under continuous write/erase voltage pulses of +1.5 V, 10 ms pulse duration and -2.0 V, 20 ms pulse durations at the read voltage of +0.05 V. (d) Retention characteristic of LRS and HRS.


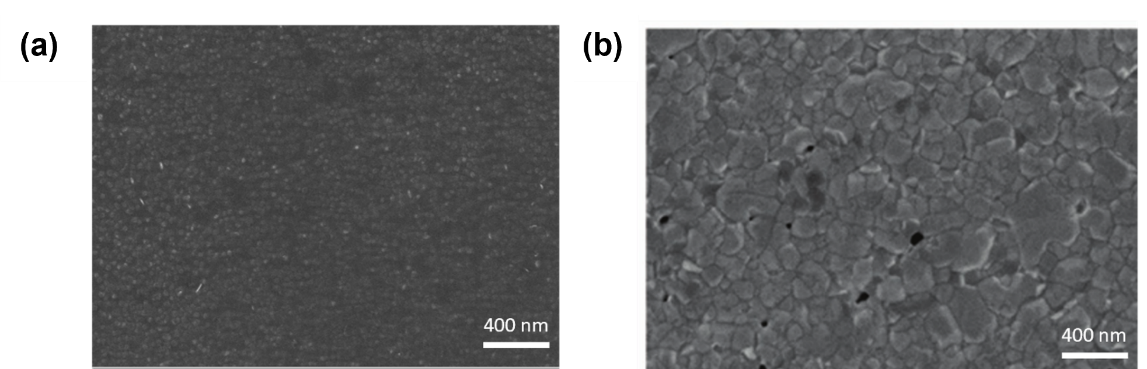


2. Comparison of bulk and quantum dot film by SEM image

Figure S2 Top-view SEM image of QDs layer (left) and bulk film (right).^1^

The working mechamism was expalined by ohmic conduction and space charge limited conduction. The detailed equation is given in the supporting informaiton.

Equation 1. Equations expaining the working mechanism of the device

$J_{ohmic}=\sigma E=q\mu N_{C}Eexp[-\frac{E_{C}-E_{F}}{kT}]$ (1)

 σ is electrical conductivity, µ is the electron mobility, *N_c_* is the effective density of states of the conduction band, *E_C_* is the conduction band and *E_F_* is the Fermi energy level.

$J_{SCLC}=\frac{9}{8}\varepsilon_{i}\mu\theta\frac{V^{2}}{d^{2}}$ (2)

ε*_i_* is the permittivity of the oxide, µ is the mobility, θ is the ratio of free and shallow trapped charge, and *d* is thickness of the film.
